# HIV voluntary counseling and testing uptake and associated factors among Ethiopian youths: evidence from the 2016 EDHS using multilevel modeling

**DOI:** 10.1186/s12879-021-06021-x

**Published:** 2021-04-09

**Authors:** Mamo Nigatu, Teshome Kabeta, Abonesh Taye, Merga Belina

**Affiliations:** 1 Faculty of Public Health, Department of Epidemiology, Jimma University, Institute of Health, Jimma, Oromia Ethiopia; 2 Faculty of Public Health, Department of Human Nutrition and Dietetics, Jimma University, Institute of Health, Jimma, Oromia Ethiopia; 3 College of Natural & Computational Sciences, Department of Statistics, Addis Ababa University, Addis Ababa, Ethiopia

**Keywords:** EDHS, Multilevel, Youths, Voluntary HIV counselling, And testing

## Abstract

**Background:**

Existing evidence showed that Human Immunodeficiency Virus counselling and testing uptake among Ethiopian youths is low, and factors contributing to it are not well studied. Therefore, this study aims to assess the status of uptake and identify its determinants using the 2016 Ethiopia Demographic and Health Survey data.

**Method:**

Data of 10,903 Ethiopian youths were extracted from the 2016 Ethiopian Demographic and Health Survey. The association between the response variable and the predictors was modeled by multilevel binary logistic regression, whereas adjusted odds ratio and confidence intervals were used to measure associations and their statistical significance. The variation in the uptake of counselling and testing of HIV across regions of Ethiopia was quantified by intra-class correlation.

**Result:**

The current study revealed that**,** overall, 34.9% (95% CI: 33.5, 36.2%) Ethiopian youths were ever tested for human immunodeficiency virus. Results show that about 9% of the variation in the probability of being tested for the disease was due to the regional variations. Moreover, having moderate and comprehensive HIV knowledge, being rich, having risky sexual behaviour, having a better educational level, having professional work, being married, owning of mobile, and having access to media were positively associated with human immunodeficiency virus voluntary counselling and testing uptake. On the other hand, being male, following protestant religion, following Muslim religion, and following other religions than orthodox religion were negatively associated with the uptake of human immunodeficiency virus counselling and testing.

**Conclusion:**

Voluntary human immunodeficiency virus counselling and testing uptake among Ethiopian youths is very low and varies across the regions which might hamper the ambitious plan of Ethiopia to end the disease as a public health threat by 2030. Emphasis should be given to promoting the youths’ HIV-related knowledge through community-based education, encouraging and empowering the youths to participate in professional works by giving due focus to poor youths, and promoting mass media utilization to better achieve the plan.

## Background

Any person who is in the age group of 14 to 24 years, according to the WHO, is considered as a youth [1]. According to the Ethiopian Federal HIV/AIDS Prevention and Control Office (HAPCO) and Federal Ministry of Health guideline, youths are among the top priority population segments for VCT which is given free of charge since they are vulnerable to the Human Immunodeficiency Virus (HIV) because of the strong influence of peer pressure and the development of their sexual and social identities which often lead to experimentation [2]. HIV, unlike many other diseases, continued to be a major challenging public health problem to prevent and control. Starting from the first occurrence of the pandemic, more than seventy-five million people have been infected by the disease, and it has claimed more than 32 million lives [3–5]. The global community is committed to an ambitious plan of bringing the acquired immunodeficiency syndrome (AIDS) to an end by the year 2030 [6]. In 2014, the United Nations Program on Acquired Immunodeficiency syndromes (UNAIDS) being with other stakeholders launched the three 90s targets of diagnosing 90 % of all HIV-positive persons, providing antiretroviral therapy (ART) for 90 % of those diagnosed, and achieving viral suppression for 90 % of those treated by the year 2020 [4]. However, according to the reports from the 2019 UNAIDS and World Health Organization (WHO), globally, 37.9 million people were living with the disease at the end of 2018, whereas, 1.7 million people and 770,000 people were respectively newly infected and died from the disease-related causes [3, 5]. Even though the global annual number of new infections and death have declined, reaching the 2020 milestone with the current achievement is unthinkable [5, 7]. The disease disproportionally affected Sub-Saharan Africa where more than 70% of the disease’s global burden has occurred. Two-third of the estimated 6000 new infections that occur globally each day occur in SSA [8]. East and Southern Africa is the most affected African region where 20.6 million people had been living with HIV and 800,000 were newly infected in 2018 [9]. The number of people living with HIV in Ethiopia was decreased from the 2016 WHO estimate of 710,000 to 690,000 in 2018 [7, 10]. However, 23,000 people were newly infected at the end of 2018 leaving Ethiopia far off achieving the 2020 target [7].

In 2017, globally, 590,000 youths were newly diagnosed with HIV disease and 3.9 million youths were living with the disease [11]. According to the evidence from 2020 WHO estimates, globally, often people who were newly diagnosed with HIV infection, three persons were youths [1]. Approximately, worldwide, one thousand six hundred youth contract HIV infection every single day, and one young person loss his/her life due to the illnesses related to AIDS every 10 min [11]. In 2018, 21 % of the total 37,832 newly diagnosed HIV cases in the US were among the youth [12]. In 2017, 290,000 youths were newly diagnosed with HIV infections in Eastern and Southern Africa which were the highest of all the diseases’ incidences that occurred among youths all over the world in the same year [11].

The risk of contracting HIV infection among youths is highly associated with age at sexual initiation. Hence, abstaining from sexual intercourse and delaying the age at sexual debut is among the efficient HIV prevention efforts for young people [1]. Studies have also witnessed that HIV-related age at sexual debut, stigma, and discrimination, place of residence, educational level, age, marital status, number of a life sex partner, exposure to mass media, Having Antenatal Care (ANC) follow up, having sexually-transmitted disease (STI) symptoms, wealth index and knowing about HIV were among other variables significantly associated with HIV Voluntary Counselling and Testing (VCT) uptake among youths [13–17]. Effectively addressing the root causes that put young people at risk of new HIV infections is an overarching intervention to achieve the ambitious plan of ending AIDS by 2030 [11]. Young people HIV counseling and testing create the gateway to care, treat, and support them [2, 18]. To affirm that young people exercise their right to know their HIV status and benefitted from antiretroviral treatment (ART), HIV testing and counseling must be radically scaled up [19].

Pieces of evidence from the 2016 Ethiopian Demographic and Health Survey (EDHS) report showed that 9% of young women and 1% of young men age 15–24 had started sexual intercourse before their 15 years of age. However, only 27% of young women and 29% of young men who had sexual intercourse in the past 12 months before the commencement of the survey was tested for HIV and received the results of the test [20]. Despite this low HIV VCT uptake among Ethiopian youths, to the best knowledge of the authors, there is no study done to address potential factors associated with it using nationally representative data. Moreover, there was also a dearth of study that tried to consider regional variations about the uptaking of youths’ HIV VCT. Therefore, the current study is aimed to determine the status of HIV VCT uptake and to identify factors associated with HIV voluntary counseling and testing among Ethiopian youths using 2016 nationally representative EDHS data.

## Methods

### Data sources

Central Statistical Agency (CSA) of Ethiopia and other stakeholders by technical assistance of ICF International collected the 2016 EDHS data; processed and organized it into different datasets. The authors have accessed the dataset from a public domain MEASUREDHS website by permission. The authors didn’t involve in any part of the sampling design and data collection. The authors used this as secondary data.

### Sampling design for the EDHS

Ethiopia is divided into 9 regions and two city administrations. The hierarchy of Ethiopian government structure has four layers namely Regions, Zones, Weredas, and Kebele in order of higher to lower level respectively. The sampling frame used in the 2016 EDHS was obtained from the Population and Housing Census (PHC) conducted in 2007 by CSA. A random and representative sample was obtained by two-stage sampling. The details of the sampling procedure are found in the 2016 EDHS report [21].

### Measurements and operational definitions

The outcome variable was a single direct question asking whether the respondent has ever been tested for HIV or not (the response was Yes/No). In the context of this study, HIV VCT means that the person has already got both counseling and testing service. A questionnaire was used to get participants response on the following set of variables; sex, age, religion, wealth status, marital status, occupational status, sex of household head, education level, place of residence and region, and other variables like coverage status of health insurance, frequency of reading newspaper or magazine in a month, frequency of listening to the radio in a month, frequency of watching television, use of the internet, frequency of using the internet within a month, decisions on personal health care, relationship with a most recent sex partner, owning of a mobile telephone, discussion about family planning with a health worker, decisions on large household purchases and decisions on how to spend respondent’s earnings, and respondent’s involvement during check-ups for the most recent child. Stigma status was measured by six domains of stigma [22]. Similarly, HIV knowledge was also measured by another six domains of its measurement [23]. Based on its six domains of measurements the knowledge of HIV is categorized as low, moderate, and comprehensive. If the scores of the six domains of HIV knowledge measurement are summed from 0 to 2 it is categorized as low level of HIV knowledge. If the sum of the response to the six domains is between 4 and 5, it is categorized as a moderate level of HIV knowledge. If the sum of the response to the six domains of HIV knowledge scores six, it is categorized as comprehensive HIV knowledge. Likewise, stigma towards HIV patients was categorized based on six domains of measurement. The youths were categorized as having no stigma towards HIV patients if the scores of the six domains of HIV stigma and discrimination summed to 0, Moderate HIV stigma if the sum of the response to the six domains is between 1 and 3, and high HIV stigma if the sum of the response to the six domains of HIV stigma and discrimination scores is between 4 and 6. On the other hand, the youths were considered as practicing risky sexual behavior if they responded either as having multiple sex partners, i.e. more than one sex partner, in a lifetime or if they had multiple sex partners in the last 12 months excluding the spouse.

In the 2016 EDHS dataset, the occupational status of the respondents was presented in too much detail and the authors have reclassified it as agricultural workers, professional workers, trade or sales workers, elementary occupation, and other workers [24]*.*

### Data analysis

Stata 14.2 statistical software was used for extracting relevant variables, data processing, summarizing descriptive data and running inferential statistics. To assess regional variation of HIV-VCT uptake among the youths and to identify its associated factors, the multilevel logistic regression model was fitted. As the surveys are based on multistage stratified cluster sampling, the DHS surveys often follow a hierarchical data structure [20]. The degree of dependency in the clustered data plays a great role in the estimation of the parameters in the analysis of multilevel regression and is captured by intra-class correlation (ICC), which reflects the proportion of total variation in the response variable which is accounted for by the between-group variation [25]. The weights used in the analysis were obtained from DHS and were adjusted as per the recommendation by Adam [26]. The goodness of fit was checked after weighting the dataset by both candidate weights. A model with the smallest AIC and BIC with its significant variables is considered, and the results are presented in Table 3.

### Ethical consideration

The Federal Democratic Republic of Ethiopia Ministry of Science and Technology and the Institutional Review Board of ICF International reviewed and approved The EDHS 2016 survey protocol. Written consent was obtained from each participant. The investigators received permission from the public domain MEASUREDHS website and reanalyzed the data set on youth respondents whose ages range from 15 to 24.

## Results

### HIV VCT uptake

**Fig. 1:**
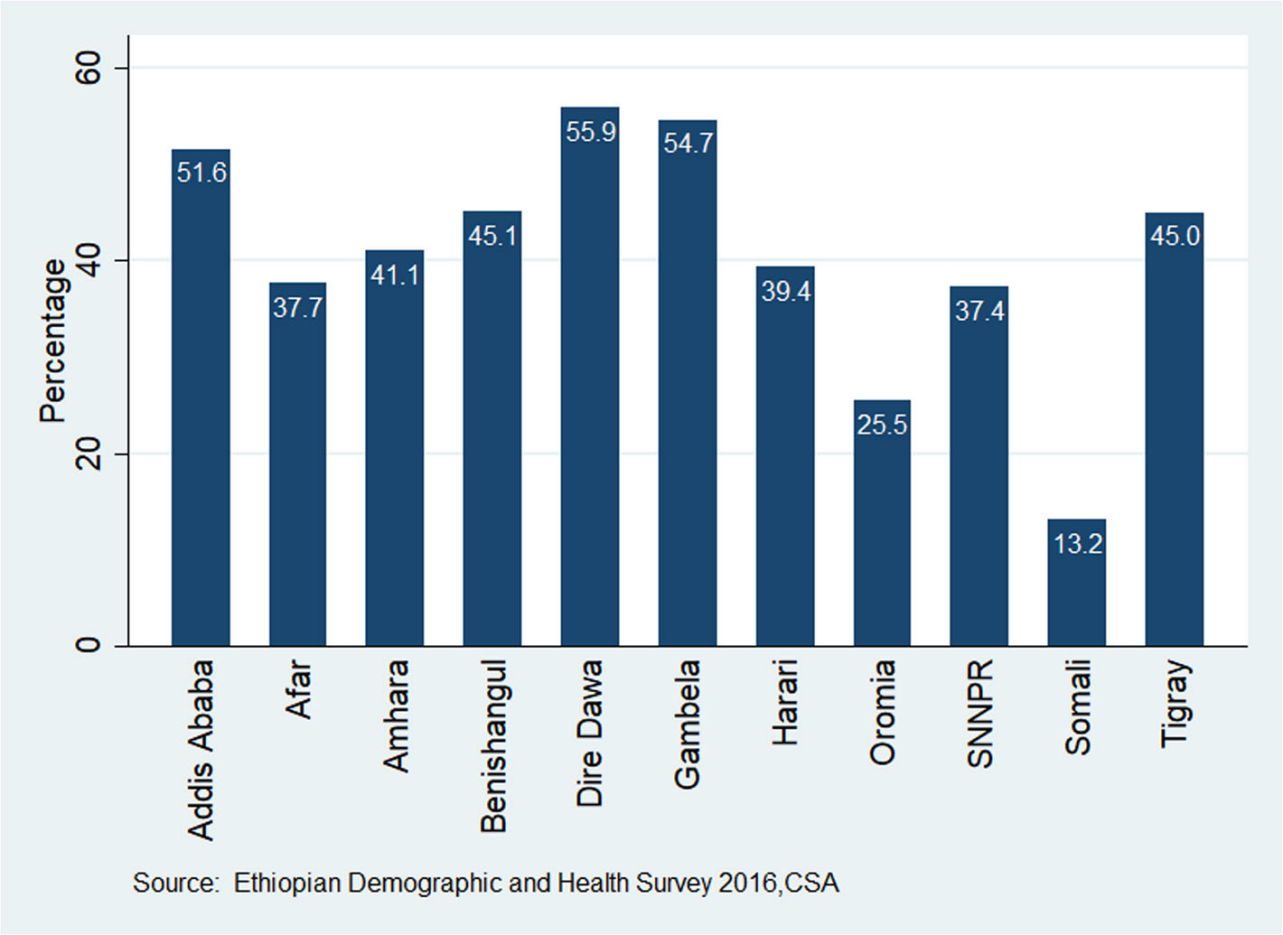
HIV VCT uptake among the Ethiopian youths by regions in the country, 2016 EDHS

The current study revealed that the overall representation of the prevalence of VCT uptake among Ethiopian youths was estimated to be 34.9% and a 95% CI [33.5, 36.0%]. Among the youths ever been tested for HIV, the youths living in Dire-Dawa special district were the leading where 55.9% of them were tested followed by Gambella and Addis Ababa youths where 54.7 and 51.6% were respectively tested while the Somali region’s youths registered the lowest testing figure where only 13.2% were tested for the disease (Fig. 1).

### Characteristics of participants

Ten thousand one hundred thirteen (10,903) Ethiopian youths participated in the 2016 EDHS. The mean age of the study participants was 19.1 (+ 2.8) years. Regarding their religion, orthodox Christian followers were the dominant religious group (43.9%) followed by Muslims (30.4%). Concerning their economic and marital status, nearly half (49.0%) of the participants were rich, and more than two-thirds (69.7%) of them were never in marital union. More than three-fourth (76.5%) of the youths who participated in the study were from male-headed households. Agriculture is the leading type of occupation constituting 33.7% of all types of work captured in the survey. The majority, more than three-fourth, (78.0%) of the youths included in the study were rural dwellers (Table 1).

**Table 1:** Socio-demographic characteristics of Ethiopian youths, EDHS 2016

Variables	Categories	Total (%)	Ever been tested for HIV			
			Yes		No	
			N	%	N	%
Religion	Orthodox	4647 (43.9)	1980	42.6	2667	57.4
	Protestant	2500 (23.6)	810	32.4	1690	67.6
	Muslim	3222 (30.4)	846	26.3	2376	73.8
	Other	299 (2.2)	57	24.9	172	75.2
Wealth Status	Poor	3450 (32.6)	835	24.2	2615	75.8
	Middle	1960 (18.5)	598	30.5	1362	69.5
	Rich	5187 (49.0)	2260	43.6	2928	56.4
Marital Status	Never in union	7388 (69.7)	2040	27.6	5349	72.4
	Married	2684 (25.3)	1363	50.8	1322	49.2
	Other	525 (5.0)	291	55.3	235	44.7
Sex of household head	Male	8104 (76.5)	2756	34.0	5348	66.0
	Female	2494 (23.5)	937	37.6	1557	62.4
Occupational Status	Not working	4262 (40.2)	1358	31.9	2905	68.2
	Agricultural Workers	3572 (33.7)	988	27.7	2584	72.3
	Professionals	253 (2.4)	199	78.8	54	21.2
	Trade/Sales	1014 (9.6)	529	52.2	485	47.9
	Elementary work	968 (9.1)	334	34.5	634	65.5
	Others	528 (5.0)	285	54.0	243	46.1
Residence	Urban	2335 (22.0)	1200	51.4	1134	48.6
	Rural	8263 (78.0)	2493	30.2	5770	69.8
Educational level	No education	1773 (16.7)	411	23.2	1362	76.8
	Primary	6076 (57.3)	1783	29.3	4294	70.7
	Secondary	2095 (19.8)	1046	49.9	1048	50.1
	Higher	654 (6.2)	453	69.3	200	30.7
Sex of the respondent	Female	6143 (58.0)	2315	37.7	3828	62.3
	Male	4455 (42.0)	1378	30.9	3077	69.1

Of all Ethiopian youths who participated in the study, 11.4% of them had risky sexual behavior, whereas, 63.2 and 30.5% of youths had moderate to a high level of stigmatized attitude towards PLWH. Regarding their knowledge of HIV, less than one-third, 30.3% of them had comprehensive HIV knowledge, whereas, one-fifth of them had low HIV knowledge. The Majority, more than two-thirds, 69.6% of them did not discuss FP with healthcare workers in the last few months before the survey. Regarding the coverage of health insurance among Ethiopian youths included in the current survey, only 5.4% of youths were covered with health insurance. From all married-youths included in the survey, 66.6 and 67.9% of youths were from households where respondents and their wives or their partners jointly decide on respondents’ health care and large households’ purchase respectively. The majority, 88.8% of the youths replied that the most recent sex partner relationship they had was a spousal relationship (Table 2).

**Table 2:** Behavioral and Individual Characteristics of Ethiopian Youths, 2016 EDHS

Variables	Categories	Total (%)	Ever been tested for HIV			
			Yes		No	
			N	%	N	%
Risky Sexual Behavior	No	9389 (88.6)	2935	31.3	6454	68.7
	Yes	1209 (11.4)	758	62.7	451	37.3
Stigma Status	No	635 (6.3)	280	44.2	355	55.8
	Moderate	6347 (63.2)	2479	39.1	3868	61.0
	High	3060 (30.5)	934	30.5	2127	69.5
Knowledge of HIV	Low	2086 (20.9)	482	23.1	1605	76.9
	Moderate	4863 (48.8)	1866	38.4	2997	61.6
	Comprehensive	3023 (30.3)	1341	44.3	1683	55.7
Discussion about FP with HWs in a month	No	1434 (69.6)	744	51.9	690	48.2
	Yes	628 (30. 5)	417	66.4	211	33.6
Covered by health insurance	No	10,028 (94.6)	3450	34.4	6578	65.6
	Yes	569 (5.4)	243	42.6	327	57.4
A person who should have a greater say: respondent’s health care	Respondent alone	308 (13.4)	186	60.2	123	39.8
	Respondent and wife/partner	1531 (66.6)	816	53.3	715	46.7
	Wife/partner alone	436 (19.0)	170	39.0	266	61.0
	Someone else	16 (0.7)	6	37.3	10	62.7
	Other	6 (0.3)	2	28.3	4	71.7
A person who usually decides large on household purchases	Respondent alone	183 (8.0)	98	53.4	85	46.6
	Respondent and wife/partner	1561 (67.9)	860	55.1	701	44.9
	Wife/partner alone	523 (22.8)	212	40.5	311	59.5
	Someone else	26 (1.13)	7	25.2	19	74.8
	Other	5 (0.2)	4	75.3	1	24.7
Relationship with the most recent sex partner	Spouse	2226 (88.8)	1156	51.9	1070	48.1
	Girlfriend/fiancé	161 (6.4)	111	68.8	50	31.2
	Casual acquaintance	11 (0. 5)	6	50.4	6	49.6
	Commercial sex worker	1 (0.1)	1	100.0	0	0.0
	Live-in partner	107 (4.3)	55	51.8	51	48.3
	Other	1 (0.03)	0	10.9	1	89.1

### The HIV VCT uptake and concomitant factors

Multilevel logistic regression was fitted to identify independent predictors of HIV VCT uptake among Ethiopian Youths. The intra-class coefficient was calculated to assess the regional variation of HIV VCT uptake among the youths. Accordingly; knowledge of HIV, wealth status, risky sexual behavior, religion, educational level, marital status, occupational status, owning a mobile phone, frequency of listening to the radio, and gender were the predictors of HIV VCT uptake among Ethiopian youths.

The odds of getting tested for HIV for Ethiopian youths who had moderate and comprehensive HIV knowledge were nearly 1.78 (AOR = 1.78, 95% CI: 1.45–2.19) and 2.16 (AOR = 2.16, 95% CI: 1.73–2.72) times higher compared to their peers who had low HIV knowledge. On the other hand, Ethiopian youths’ wealth status was also significantly associated with their HIV test uptakes. The odds of getting HIV tests for Ethiopian youths with middle and rich wealth status were nearly 1.28 (AOR = 1.28, 95%CI: 1.07–1.53) and 1.72 (AOR = 1.72, 95%CI: 1.39–2.14) times higher compared to their poor peers. The odds of getting HIV tests for youths who had risky sexual behavior were about 2.39 (AOR = 2.39, 95%CI: 2.04–2.80) times higher compared to youths who had no risky sexual behavior. Ethiopian youths’ religions were also independently predicted their HIV test uptake; for Protestant, Muslim, and other religions follower youths, the odds of getting HIV test were lower by 23% (AOR = 0.77, 95%CI: 0.63–0.95), 22% (AOR = 0.78, 95%CI: 0.66–0.91) and 34% (AOR = 0.66, 95%CI: 0.53–0.82) compared to Orthodox Christians youths, respectively.

For Ethiopian youths who completed primary, secondary and higher education the odds of getting HIV test was 1.46 (AOR = 1.46, AOR = 1.13–1.87), 2.47 (2.47, 95%CI: 1.85–3.29), and 3.49 (AOR = 3.49, 95%CI: 2.57–4.73) times higher compared to their Ethiopian uneducated peers. The odds of getting HIV tests for married youths and youths with other marital status were nearly 5.37 (AOR = 5.37, 95%CI: 3.72–7.74) and 4.60 (AOR = 4.60, 95%CI: 3.41–6.21) times higher compared to their peer youths who are not in marital union. For professional worker youths, trade/sales worker youths, and youths who were engaged in other occupations, the odds of getting HIV test were 1.87 (AOR = 1.87, 95%CI = 1.14–3.07), 1.45 (AOR = 1.45, 95%CI: 1.03–2.06) and 1.60 (AOR = 1.60, 95%CI: 2.20–2.13) times higher compared to their Ethiopian peer youths who were not engaged in any kind of occupation. Regarding mobile telephone ownership, the odds of getting an HIV test for mobile-owner Ethiopian youths was 1.88 (AOR = 1.88, 95%CI: 1.61–2.20) times higher compared to their Ethiopian peers who did not own mobile. Concerning access to media, the odds of getting an HIV test for Ethiopian youths who listen to the radio at least once a week was 1.33 (AOR = 1.33, 95%CI: 1.12–1.57) times higher compared to their Ethiopian peers who did not listen to the radio. Likewise, the odds of getting HIV tests for male Ethiopian youths were lower by 30% (AOR = 0.70, 95%CI: 0.60–0.83) compared to their female counterparts.

The result of ICC showed that 9.0% (±4.7%) of variation in the likelihood of HIV testing uptake among the Ethiopian Youths was explained by the regional variation which was statistically significant (95% CI:3.1–23.4%). This indicates that estimating HIV testing uptake among Ethiopian youths at a national level would have resulted in a biased estimate had we had not considered the regional variation. The median odds ratio (MOR) was estimated to be 1.94 (Table 3).

**Table 3:** Results from Multilevel Binary Logistic Regression Using Data from Ethiopians Youths, 2016 EDHS

VCT uptake	Robust Std. Err.	Z	P > z	AOR [95% Conf. Int.]
**Knowledge of HIV** (Ref.:Low)				
Moderate	0.190	5.4	0.0000	1.78 [CI (1.45, 2.19)]
Comprehensive	0.249	6.7	0.0000	2.16 [CI (1.73, 2.71)]
**Wealth Status** (Ref.: Poor)				
Middle	0.114	2.8	0.0050	1.28 [CI (1.07, 1.53)]
Rich	0.189	5	0.0000	1.72 [CI (1.39, 2.14)]
**Risky Sexual Behavior** (Ref.: No)				
Yes	0.195	10.7	0.0000	2.39 [CI (2.04, 2.80)]
**Religion** (Ref.: Orthodox)				
Protestant	0.081	−2.5	0.0130	0.77 [CI (0.63, 0.95)]
Muslim	0.061	−3.2	0.0010	0.78 [CI (0.66, 0.91)]
Other	0.073	−3.8	0.0000	0.66 [CI (0.53, 0.82)]
**Educational level** (Ref: No Education)				
Primary	0.186	3	0.0030	1.46 [CI (1.13, 1.87)]
Secondary	0.360	6.2	0.0000	2.47 [CI (1.85, 3.29)]
Higher	0.542	8	0.0000	3.49 [CI (2.57, 4.73)]
**Marital Status** (Ref.: Never in a union)				
Married	1.004	9	0.0000	5.37 [CI (3.72, 7.74)]
Other	0.704	10	0.0000	4.60 [CI (3.41, 6.21)]
**Occupational Status** (Ref.: Not working)				
Agricultural Workers	0.092	−0.6	0.5640	0.95 [CI (0.78, 1.14)]
Professional Workers	0.473	2.5	0.0130	1.87 [CI (1.14, 3.07)]
Trade/Sales	0.260	2.1	0.0360	1.45 [CI (1.03, 2.06)]
Elementary occupation	0.194	0.6	0.5640	1.11 [CI (0.78, 1.56)]
Others	0.233	3.2	0.0010	1.60 [CI (1.20, 2.13)]
**Own a mobile telephone**				
Yes	0.151	7.9	0.0000	1.88 [CI (1.61, 2.20)]
**Freq. of listening to a radio (**Ref.: No)				
Less than once a week	0.070	1.7	0.0860	1.11 [CI (0.98, 1.26)]
At least once a week	0.114	3.3	0.0010	1.33 [CI (1.12, 1.57)]
**Sex of the youths (**Ref.: Female)				
Male	0.057	−4.3	0.0000	0.70 [CI (0.60, 0.83)]
_cons	0.017	−11.4	0.0000	0.07 [CI (0.05, 0.12)]
**Region**				
var.(_cons)	0.044			0.14 [CI (0.75, 0.26)]

*AOR* Adjusted Odds Ratio

## Discussion

Nearly one-third, 34.9% of the Ethiopian youths have ever got HIV voluntary counseling and testing. This finding is almost consistent with the finding from the study done in Sub-Saharan Africa using secondary data analysis of DHS where 36.5% of youths were ever tested for HIV [27]. However, the current finding is lower than the findings from the studies done in Uganda, South Africa, and Tanzania where 81.8, 52.2, and 40% of youths were ever tested for HIV [28–30] and is greater than the finding from Nigeria where less than one-fourth (23.7%) of youths were ever tested for HIV [31]. The differences could be attributed to differences in awareness and knowledge of HIV between youths residing in different countries.

In the current study, Youths’ HIV knowledge was independently associated with their HIV VCT uptake. The odds of getting HIV tests for Ethiopian youths who had moderate and comprehensive HIV knowledge were nearly 1.78 times higher compared to their peers who had low HIV knowledge. This finding is consistent with the results from the study done using data from four Sub Saharan countries: Burkina Faso, Senegal, South Africa, Southeast Asia, and Kenya, where youths who had higher knowledge of HIV opted in HIV test uptake [27, 29, 32–36]. This might be due to the reason that people with better HIV knowledge have less misconception on the disease and less stigmatized attitude towards PLWH and have higher odds of being tested for HIV. Studies have witnessed that people with comprehensive knowledge of HIV have a less stigmatized attitude towards people living with HIV [37–40].

On the other hand, Ethiopian youths’ wealth status was also significantly associated with their HIV test uptakes. The odds of getting HIV tests for Ethiopian youths with middle and rich wealth status were nearly 1.28 and 1.72 times higher compared to their poor peers. This finding is similar to the results of many other studies done in Southeast Asia and Africa where youths from households in the highest wealth quintile were more likely to opt-in for HIV testing as compared to youths from households in the poor wealth quintile [31, 33, 41, 42]. This can be explained by differences in access to media and access to health facilities for rich people than poor people. On the other hand, many studies have witnessed that exposure to mass media promotes HIV-related knowledge and better creates awareness on HIV [43–51]. The current study and many other aforementioned studies witnessed that HIV-related knowledge boosts HIV test uptake.

The odds of accepting HIV test among youths who had risky sexual behavior was about 2.4 times more likely than the odds of accepting HIV test among youths who had no risky sexual behavior. This finding is in line with the studies done in southeast Asia, China, Burkina Faso, and Cambodia where youths with higher risk sexual behavior received HIV testing more likely than youths with low risky sexual behavior [32, 34, 41, 52]. This could be due to the perceived risk of being infected for people who have risky sexual behavior. An exploratory qualitative study done in Kampala, Uganda showed a low perceived risk of HIV infection is associated with low HIV test uptake among youths [53].

Ethiopian youths’ religions were also independently predicted their HIV test uptake; for Protestant, Muslim and other religion follower youths, the odds of getting HIV test were lower by 23.0, 22.0, and 34.0% compared to Orthodox Christian youths respectively. This finding supports the finding from the study done among reproductive age Ethiopian women where Christian and Muslim women were less likely to accept HIV testing as compared to Orthodox Christian women [54]. The study in Tanzania among Catholic, Lutheran, and Pentecostal churches parishioners also showed that shame-related HIV stigma is strongly associated with religious beliefs such as the belief that HIV is a punishment from God or that people living with HIV/AIDS (PLWHA) have not followed the Word of God [55]. This could be due to the differences in beliefs and dogmas across different religions.

The odds of getting HIV tests for Ethiopian youths who completed primary, secondary, and higher education were 1.45, 2.47, and 3.49 times higher compared to their Ethiopian uneducated peers. This finding supports the findings from secondary data analysis of DHS which were done in Cambodia, Burkina Faso, Malawi, Tanzania, and Senegal where academic advancement was highly associated with receiving HIV testing [30, 32, 33, 41, 56]. This could be due to the reason that educated youths have better HIV knowledge and have less stigmatized attitudes towards PLWH. The current study has shown that youths who have deep insight into HIV better accept HIV testing.

For married youths and youths with another form of marital status, the odds of getting HIV tests were nearly 5.37 and 4.60 times higher compared to their peer youths who are not in marital union. Studies from Cambodia, Malawi, Tanzania, and Senegal using secondary DHS data analyses also showed that ever-married women more likely accept HIV testing as compared to never-married women which are consistent with the findings from the current study [30, 33, 41, 56]. There could be different possible reasons for this. Married youths seek health services, especially services related to pregnancy, and maybe counseled to receive HIV testing by their health care providers. On the other hand, married youth may also seek HIV counseling and testing services if they doubt their partner’s HIV serostatus. Married youths may also make joint-decisions to seek HIV counseling and testing to safeguard their marriage and the health of their current or future child/children.

Regarding the occupational status, professional worker youths, trade/sales worker youths, and youths who were engaged in other occupations were 1.87, 1.45, and 1.60 times more likely to accept the voluntary HIV testing and counseling compared to their Ethiopian peer youths who were not engaged in any kind of occupation. The study in rural Ethiopia also showed that people who are engaged in professional work were more likely to accept HIV testing compared to their peer farmers [57]. However, the study done in Guizhou province, China showed that farmers were more likely to receive HIV voluntary counseling and testing services than other professionals [52]. The discrepancy could be due to differences in HIV-related awareness between farmers in developing and developed countries.

Regarding mobile telephone ownership, the odds of getting an HIV test for mobile owner Ethiopian youths was 1.88 times higher compared to their Ethiopian peers who did not own mobile. The finding is in line with the finding of the study done in Senegal using the secondary data analyses of DHS data where women and men mobile owners were respectively 40 and 90% more likely to accept HIV testing services as compared to non-mobile owners [33]. Ethiopian youths who listen to the radio at least once a week were 1.33 times more likely to accept HIV voluntary counseling and testing compared to their Ethiopian peers who did not listen to the radio. The findings from secondary data analysis of DHSs data from 27 SSA countries and DHS data from India showed that people who frequently watch television and frequently listen to the radio had more comprehensive HIV knowledge than people who do not [45, 47]. Many other studies [29, 44, 46, 48, 49, 51] had also witnessed that exposure to mass media has an independent impact on acquiring comprehensive HIV knowledge. The current study and other aforementioned studies have witnessed that better knowledge of HIV was associated with better HIV counseling and testing uptake.

For male Ethiopian youths, the odds of getting tested for HIV was lower by 30% compared to their female counterparts. This finding is consistent with the finding from the study done in Sub Saharan Africa using secondary data analysis of DHS where the odds of getting tested for HIV among male youths was 68% less likely than the odds of getting tested among their female peers [27] and the study in South Africa where female Youths more likely accepted HIV testing than their male peers [29]. This could be due to the less risk perception and the reluctance of males to seek HIV counseling and testing. The gender dynamics and perceptions of HIV testing study done in Lesotho showed that males usually say HIV testing is for females [58]. On the other hand, females do have a high probability of getting HIV counseling and testing during the antennal follow-up during pregnancy and during postnatal care after child delivery.

The intra-class correlation was used to capture the magnitude of variability concerning the prevalence of VCT uptake across the regions in Ethiopia. The result of ICC showed that 9.0% (±4.7%) of variation in the likelihood of HIV testing uptake among the Ethiopian Youths was explained by the regional variation which was statistically significant. This indicates that estimating HIV testing uptake among Ethiopian youths at a country level would have resulted in a biased estimate had we had not considered the regional variation. From the multilevel model it was estimated that if a youth moved to another region with a higher probability of HIV testing uptake, the median increase in their odds of HIV testing uptake would be 1.94-fold (MOR = 1.94).

The current study has its strengths and limitations. The EDHS data is the most reliable data as the survey method was methodologicaly rigorious. A large number of Ethiopian youths were proportionally selected from every region of the country to ensure national representation of the youths who were included in the study. To minimize bias that could have been introduced as a result of clustering effects, the authors weighted the DHS data before conducting further analyses. To account for the variation of levels of HIV counseling and testing across the regions in the country, moreover, multilevel logistic regression modeling was done.

On the other hand, the current study does not assure the temporal relationship between HIV test uptake among Ethiopian youths and its predictors as data related to both the outcome and predictor variables were simultaneously collected, and the yielded evidence should be utilized cautiously. Furthermore, due to the absence of qualitative data from the 2016 EDHS, the investigators failed to investigate the link between socio-cultural factors and HIV test uptake among the youths. Lastly, the only variable we have at the community level was ‘region’ for which intraclass correlation was reported.

## Conclusions

Regardless of all efforts put in a place to prevent HIV/AIDS in Ethiopia since the beginning of the disease in the country, still only about one-third (34.9%) of Ethiopian Youths have ever tested for HIV and know their HIV serostatus. The study also revealed that there are significant disparities in HIV voluntary counseling and testing uptake among Ethiopian youths living in different regions of the country. Youths who have less HIV knowledge, who are from poor households, who do not have risky sexual behavior, who are protestant Christian and Muslims, who are male, who are less educated, who are not in marital union, who are not professional workers, who do not listen to the radio and who do not have a mobile phone are less likely to accept HIV counseling and testing as compared to their counterpart peers. One of the strategies launched by UNAIDS and other partners to control HIV was, diagnosing 90% of people living with HIV by 2020. The Democratic Republic of Ethiopia has also committed to ending AIDS as a public health threat by 2030. But with the current unacceptable lower magnitude of HIV counseling and testing uptake among Ethiopian youths, achieving this target is unthinkable. Therefore, any stakeholder working on HIV prevention and control in the country should give due emphases to promoting youths’ academic advancement by integrating and streamlining HIV-related education in academic curricula, expanding mobile and other media coverages, promoting HIV-related awareness creation through community-based educations and religious organizations, and encouraging and strengthening youths to participate in professional works to boost the current unacceptably low level of VCT among them to meet the ambitious plan of ending HIV as a public health threat by 2030.

## Data Availability

The Stata format datasets used and/or analyzed during the current study are available from the corresponding author on reasonable request.

## Abbreviations

AIDS: Acquired immune deficiency syndrome
AOR: Adjusted Odds Ration
ART: Anti-Retroviral Therapy
CI: Confidence Interval
EA: Enumeration Area
EDHS: Ethiopian Demographic and Health Survey
FP: Family Planning
HIV: Human Immunodeficiency Virus
HW: Health Workers
ICC: Intra class correlation
MOR: Median Odds Ratio
OR: Odds Ratio
PLWHIV: People living with HIV
PPS: Probability Proportionate to Size
PPS: Probability Proportionate to the Size
SD: Standard Deviation
TV: Television
UNAIDS: United Nations Program on HIV/AIDS
VCT: Voluntary Counseling and Testing
WHO: World Health Organization
